# High prevalence of direct repeat unit types of 10di, 8 h and 8i among methicillin resistant *Staphylococcus aureus* strains with staphylococcal cassette chromosome *mec* type IIIA isolated in Tehran, Iran

**DOI:** 10.1186/s13756-019-0501-y

**Published:** 2019-03-06

**Authors:** Mitra Motallebi, Fereshteh Jabalameli, Reza Beigverdi, Mohammad Emaneini

**Affiliations:** 1 Infectious Diseases Research Center, Kashan University of Medical Sciences, Kashan, Iran; 2 Department of Immunology and Microbiology, Faculty of Medicine, Kashan University of Medical Sciences, Kashan, Iran; 3 Department of Microbiology, School of Medicine, Tehran University of Medical Sciences, Building No. 7, 100 Poursina St., Keshavarz Blvd., Tehran, 14167-53955 Iran

**Keywords:** Methicillin-resistant *Staphylococcus aureus*, SCC*mec* typing, *Dru* type, Virulence factor

## Abstract

**Background:**

The emergence of methicillin-resistant *Staphylococcus aureus* (MRSA) is a main concern in burn care centers worldwide. The some reports of MRSA in Iran suggested that MRSA with type SCC*mec* III is common among burn patients. The aim of this study was to determine the prevalence, virulence genes, and antimicrobial susceptibility of the direct repeat units (*dru*) types of MRSA with SCC*mec* IIIA isolated from burn wounds in a burn care center in Tehran, Iran.

**Methods:**

In total, 165 *S. aureus* isolates were collected from clinical samples. In order to detect MRSA isolates, the *mec*A gene was amplified through the polymerase chain reaction (PCR) method. Antimicrobial susceptibility was tested using the disc agar diffusion test. Moreover, the PCR method was applied to determine SCC*mec* types, virulence genes, and antimicrobial resistance genes. The *dru* region was sequenced and thereby, *dru* types and *dru* repeats were identified. A similarity matrix was used to create minimum spanning tree (MST).

**Results:**

The prevalence of MRSA was 69% (114 out of 165 isolates). Most of MRSA isolates (61 out of 114, 53.5%) were SCC*mec* type IIIA. All MRSA isolates were vancomycin-susceptible and more than 68% of MRSA isolates with SCC*mec* type IIIA were mupirocin resistant. The successful *dru* typing of isolates with SCC*mec* type IIIA revealed fourteen different *dru* types. There were two new *dru* types, namely dt10di and dt7aj. MST analysis indicated the presence of the three clusters of dt10di (cluster I), dt8i-dt8 h (cluster II), and dt11c-dt10ao-dt11dd-dt11a-dt10a (cluster III). There were significant differences between clusters I and II respecting antimicrobial resistance pattern and virulence genes.

**Conclusion:**

Three main *dru* clusters are prevalent in the study setting. The main *dru* types in the setting are dt10di, dt8i, and dt8 h. *Dru* typing can be used to differentiate MRSA strains with SCC*mec* IIIA.

## Background

*Staphylococcus aureus* (*S. aureus*) is one of the most common causes of infection among patients with severe burn wounds [1, 2]. Colonization of burn wounds with *S. aureus* can cause septicemia and substantially increase mortality rate [3]. The pathogenicity of *S. aureus* strains depends on different virulence factors such as Panton-Valentine leukocidin (PVL), staphylococcal enterotoxins, and hemolysins alpha, beta, gamma, and delta [4].

One of the major concerns in the treatment of infections is antimicrobial resistance, particularly to methicillin [5, 6]. Methicillin resistance is encoded by the *mec*A gene. In 2011, a second gene, *mec*C has been discovered that also causes methicillin/beta-lactam resistance. Both genes are situated on large, potentially mobile genetic elements, so-called SCC*mec* elements (staphylococcal cassette chromosome *mec*) [7, 8]. So far, thirteen main types of SCC*mec* have been identified [9]. These types differ from each other in size and genetic composition. Some studies reported that MRSA with SCC*mec* III is the most prevalent type of *S. aureus* in burn care centers in some countries of the world [2, 10–12]. The few reports of MRSA in Iran also suggested that MRSA with type SCC*mec* III is common among burn patients [13, 14]. *mecC* MRSA represent a recently recognised form of MRSA, encoding a divergent *mec* gene, which can colonise and cause disease in humans and a wide range of other host species [8].

A variable number of tandem repeats region including 40-bp of direct repeat units (*dru*) has been detected downstream to the *mec*A gene close to IS431 in the SCC*mec* element. The sequencing of this region can be used for detecting and subtyping methicillin-resistant *S. aureus* (MRSA) [15, 16]. A study reported that the *dru* typing of ST239 MRSA isolates provided the clearest distinction between SCC*mec* IIIA and III isolates [17]. *Dru* types are stable enough and hence, can be used in epidemiological analyses [16, 18].

Despite the high prevalence of MRSA with SCC*mec* III in burn care centers, there is limited information about its *dru* types. Therefore, the present study was conducted in a burn care center in Tehran, Iran, to determine the prevalence, virulence genes, and antimicrobial susceptibility of the *dru* types of MRSA with SCC*mec* IIIA isolated from burn wounds.

## Materials and methods

### Bacterial isolates

In total, 165 non-duplicate *S. aureus* isolates were collected using sterile swab from burn wound infections in a burn care center in Tehran, Iran. Sampling was done in four consecutive trimesters from June 2013 to June 2014. Isolates were primarily identified as *S. aureus* based on colony morphology, Gram staining, and catalase, coagulase, mannitol fermentation, and deoxyribonuclease tests [19]. Then, the identity of *S. aureus* isolates was confirmed through the amplification of the *fem*A gene based on the polymerase chain reaction (PCR) method and using primers explained in an earlier work [20]. After that, in order to identify MRSA isolates, the *mec*A gene was detected using specific primers [21, 22]. Finally, MRSA isolates were subjected to further testing.

### Antimicrobial susceptibility tests

The antimicrobial susceptibility of MRSA isolates was tested via the disc diffusion method on Mueller-Hinton agar based on the guideline recommended by the Clinical and Laboratory Standards Institute (CLSI) [23]. The discs used in this study were cotrimoxazole 25 μg, erythromycin 15 μg, clindamycin 2 μg, mupirocin 5 μg, rifampin 5 μg, linezolid 30 μg, and quinupristin-dalfopristin 15 μg (MAST, Merseyside, England). The microbroth dilution method was also used to determine the antimicrobial minimum inhibitory concentration (MIC) of oxacillin and vancomycin (Sigma, Steinheim, Germany). The control strain was *S. aureus* ATCC 29213. Moreover, the PCR method was employed to amplify the *erm*A, e*rm*C, *bla*Z, and *mup*A genes using specific primers [24].

### SCC*mec* typing and detection of virulence genes

SCC*mec* types were determined through the Multiplex-PCR as described elsewhere [21, 25]. The PCR method was used to detect the genes encoding haemolysins (*hla, hlb*), toxic shock syndrome toxin (*tst*), exfoliative toxin A (*eta*), staphylococcal enterotoxins (*sea, seb* and *sec*), and Panton–Valentine leukocidin (*pvl*) among isolates with SCC*mec* type III [22, 26, 27].

### *Dru* typing

*Dru* region was detected using the primers HVR1:59 ACTATTCCCTCAGGCGTCC 39 and HVR2:59 GGAGTTAATCTACGTCTCATC 39 [28]. The sequencing of all PCR products was performed on both strands through the same primers used in the primary PCR. The ChromasPro software (Technelysium Pty, Australia) was employed to analyze and align sequences. New repeats were confirmed through re-sequencing. Then, the nomenclature published by Goering et al. [16] (available at www.dru-typing.org) was used to detect and name *dru* repeats (dr, 40 bps) and *dru* types (dt, the combination of *dru* repeats). A minimum spanning tree (MST) was also created via the BioNumerics software v. 7.6.1 (Applied Maths, Austin, USA) and distance intervals were created using a bin distance of 1.0%. Clustering was done based on the distances among *dru* types. Accordingly, *dru* types, separated by a single MST distance, were considered to be closely related to each other and hence, were assigned to an identical cluster.

### Data analysis

Data were presented using the measures of descriptive statistics. Moreover, the Fisher’s exact test was conducted for categorical comparisons. The level of significance was set at less than 0.05.

## Results

Among 165 *S. aureus* isolates, 114 (69%) were MRSA. Most of MRSA isolates (61/114; 53.5%) were SCC*mec* type IIIA. Also, twenty (17.5%) were identified as SCC*mec* type V, two (1.7%) as SCC*mec* type I, and 31 (27.2%) as non-typable.

All MRSA isolates showed susceptibility to vancomycin (MIC_50_ ≤ 1 μg/ml, MIC_90_ ≤ 2 μg/ml), while most MRSA isolates (68%) were resistant to mupirocin.

All MRSA isolates were resistant to cefoxitin and more than 73% of them were resistant to erythromycin and clindamycin. Moreover, around 53% of MRSA isolates were resistant to mupirocin and trimethoprim-sulfamethoxazole. However, only a few of MRSA isolates showed resistance to rifampin (22%), quinupristin/dalfopristin (2%), and linezolid (2%). The MIC of oxacillin in 100% of MRSA isolates was higher than 64 μg/mL and all of isolates were susceptible to vancomycin. The most prevalent antimicrobial resistance gene was *blaZ* which was found in 85% of isolates followed by *ermA*, *mup* and *ermC* which were found in 65, 64 and 57% of isolates, respectively. The most prevalent genes encoding virulence factors in MRSA isolates were *hla* (61%), *hlb* (44%), *sea* (23%) and *seb* (2%), respectively. The *sec*, *eta*, *tst*, and *pvl* genes were not detected in any of MRSA isolates in this study.

As Table 1 shows, all *dru* types of SCC*mec* type III were successfully identified, which included fourteen different *dru* types with fifteen *dru* repeats. Among the identified *dru* types, two were new (dt10di and dt7aj). The most prevalent *dru* types among SCC*mec* type IIIA isolates were dt10di, dt8 h, and dt8i. Each minor *dru* type was observed only in one isolate.

**Table 1:** Antimicrobial resistance pattern, antibiotic resistance genes, virulence genes, and *dru* types in MRSA isolates with type III SCC*mec*

Sampling Time	Sample no.	*Dru* type	Cluster	Antibiotic resistance	Antibiotic resistance genes	Virulence genes
First trimester	1	10di	I	E, CD, TS, MUP	*ermA, ermC, blaZ*	*sea*
	2	10di	I	E, CD, TS, MUP	*ermC, blaZ*	*hla, sea*
	3	11dd	III	E, CD, TS, SYN, MUP	*ermA, mup*	*hla, hlb*
	4	10di	I	E, CD, TS, MUP	*ermA, ermC, blaZ, mup*	–
	5	7aj	–	E, CD, TS, MUP	*ermA, ermC, blaZ*	*hla, sea*
	6	10di	I	E, CD, TS, MUP	*ermA, ermC, blaZ, mup*	*hla, sea*
	7	10di	I	E, CD, TS, MUP	*ermA, ermC, blaZ, mup*	*hla, sea*
	8	14 k	–	E, CD, TS, MUP	*ermC, blaZ*	*sea*
	9	11c	III	E, CD, TS, MUP	*ermA, ermC, blaZ, mup*	–
	10	10di	I	E, CD, TS, MUP	*ermA, ermC, blaZ*	*hla, hlb, seb*
	11	10di	I	E, CD, TS, MUP	*ermA, ermC, blaZ*	*hla, seb*
	12	11a	III	E, TS, MUP	*blaZ, mup*	–
	13	5 k	–	E, CD, TS, MUP	*ermA, ermC, blaZ, mup*	–
	14	10di	I	E, CD, TS, MUP	*ermA, blaZ, mup*	*sea*
	15	10di	I	E, CD, TS, MUP	*ermA, ermC, blaZ, mup*	*hla, sea*
	16	10di	I	E, CD, TS, MUP	*ermA, ermC, blaZ*	–
	17	10di	I	E, CD, TS, MUP	*ermA, ermC, blaZ, mup*	*hla, sea*
Second trimester	18	10di	I	E, CD, TS, MUP	*ermC*	–
	19	10di	I	E, CD, TS, MUP	*ermA, ermC, blaZ, mup*	–
	20	10di	I	E, CD, TS, MUP	*ermA, ermC, blaZ, mup*	*hla, sea*
	21	10di	I	E, CD, TS, MUP	*blaZ*	*sea*
	22	10di	I	E, CD, TS, MUP	*ermA, ermC, blaZ, mup*	*hla, sea*
	23	10di	I	E, CD, TS, MUP	*ermA, ermC, mup*	*hla, sea*
	24	10di	I	E, CD, TS, MUP	*ermC, blaZ*	*hla, sea*
	25	10di	I	E, CD, TS, MUP	*ermA, blaZ*	*Sea, seb*
	26	10ao	III	E, CD, MUP	*ermA, ermC, mup*	–
	27	10di	I	E, CD, TS, MUP	*ermA, ermC, blaZ, mup*	*hla, hlb, sea*
	28	10di	I	SYN, MUP	*ermC, blaZ, mup*	*sea*
	29	10di	I	E, CD, TS, MUP	*ermA, ermC, blaZ, mup*	*hla, sea*
	30	10di	I	E, CD, TS, MUP	*ermA, ermC, blaZ, mup*	*hla*
	31	10di	I	E, CD, TS, MUP	*ermA, ermC, blaZ*	*sea*
	32	10di	I	E, CD, TS, MUP	*ermA, ermC, blaZ, mup*	*hla, sea*
	33	8af	–	E, CD, RP, MUP	*ermC, blaZ, mup*	–
	34	10di	I	E, CD, TS, MUP	*ermA, ermC, blaZ, mup*	*hla, sea*
Third trimester	35	10di	I	E, CD, TS, MUP	*ermC, blaZ*	–
	36	10di	I	E, CD, TS, MUP	*ermA, ermC, mup*	–
	37	10di	I	E, CD, TS, MUP	*ermA, ermC, blaZ, mup*	*hla, sea*
	38	10di	I	E, CD, TS, MUP	*ermA, ermC, blaZ, mup*	*hla, hlb, sea*
	39	10di	I	E, CD, TS, MUP	*ermA, ermC, blaZ*	*hla, hlb, sea*
	40	10a	III	MUP	*ermA, ermC, blaZ, mup*	*hla*
	41	10di	I	E, CD, TS, MUP	*ermC, blaZ, mup*	*seb*
	42	10di	I	E, CD, TS, SYN, LZD, RP, MUP	*ermA, ermC, blaZ, mup*	–
	43	10di	I	E, CD, TS, MUP	*ermA, ermC*	*sea*
	44	10di	I	E, CD, TS, MUP	*ermA, ermC*	–
	45	10di	I	E, CD, TS	*ermA, ermC, blaZ, mup*	*hla, hlb*
	46	10di	I	E, CD, TS, MUP	*ermA, ermC, blaZ, mup*	*hla, sea*
	47	10di	I	E, CD, TS, MUP	*ermA, ermC, blaZ*	*hla, sea*
	48	10di	I	E, CD, TS, MUP	*mup*	–
	49	10c	–	E	*blaZ*	*sea*
Forth trimester	50	8i	II	E, TS, MUP	*ermA, ermC, blaZ, mup*	*hla, hlb*
	51	8 h	II	–	*blaZ, mup*	*hla*
	52	6d	–	–	*ermA, blaZ, mup*	*hla, hlb*
	53	8i	II	CD	*ermA, ermC, mup*	*hla, hlb*
	54	8i	II	E, CD, TS, MUP	*ermA, ermC, blaZ*	*hla, hlb, sea*
	55	8 h	II	E, TS, RP, MUP	*blaZ*	*hlb, sea*
	56	8 h	II	E, CD, TS, RP, MUP	*ermA, ermC, blaZ, mup*	*sea*
	57	8 h	II	E, CD, TS, RP, MUP	*ermC*	–
	58	10di	I	E, CD, TS, MUP	*ermA, ermC, blaZ, mup*	–
	59	8i	II	E, CD, TS, RP, MUP	*ermA, ermC, blaZ*	*hla, hlb*
	60	10di	I	E, CD, TS, MUP	*ermA, ermC, blaZ, mup*	–
	61	8 h	II	E, CD, TS, RP, MUP	*ermC*	–

FOX: Cefoxitin, E: Erythromycin, CD: Clindamycin, TS: trimethoprim/ Sulfamethoxazole, RP: Rifampicin,

SYN: quinupristin/dalfopristin, LZD: Linezolid, MUP: Mupirocin

**Fig. 1:**
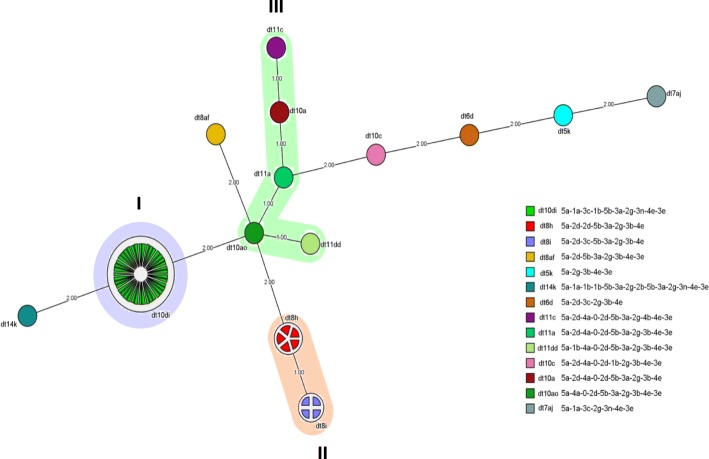
Minimum Spanning Tree generated using the BioNumerics software representing the fourteen *dru* types and the fifteen *dru* repeats observed in the studied isolates. Numerical values on the branches indicate the similarities (MST distance) between different *dru* types. BioNumerics software created similarity values (termed bins) and converted these values into distance units. The bin unit distance was set at 1% (i.e.*, dru* types at a distance of 1 on the MST had a similarity of more than 99%, while *dru* types at a distance of 2 had a similarity of 98–99%, and so on). *Dru* types were assigned to the same cluster (depicted in color) if they were separated by an MST distance of 1 (i.e. if they showed a similarity of at least 99%)

MST analysis revealed three clusters of SCC*mec* type IIIA, namely dt10di (cluster I), dt8i-dt8 h (cluster II), and dt11c-dt10ao-dt11dd-dt11a-dt10a (cluster III) (Fig. 1). Analysis of *dru* types indicated that cluster I was the most prevalent *dru* cluster in the first nine months of the study (i.e. from June 2013 to February 2014), while cluster II was the most prevalent cluster in the last trimester of the study (i.e. from March to May 2014) (Table 1).

The results of antimicrobial susceptibility tests on clusters I and II indicated that resistance to rifampin in cluster II was significantly higher than cluster I (*P* = 0.003). Moreover, the results of virulence gene analysis illustrated the significantly higher prevalence of the virulence gene *hlb* in cluster II compared with cluster I (*P* = 0.01).

Analysis of antimicrobial susceptibility pattern indicated that 92% of isolates in cluster I were resistant to erythromycin, clindamycin, cotrimoxazole, and mupirocin. On the other hand, 44% of isolates in cluster II showed resistance to erythromycin, clindamycin, cotrimoxazole, rifampin, and mupirocin.

Analysis of the patterns of antimicrobial resistance gene also showed that the most prevalent patterns in clusters I and II were *ermA* + *ermC* + *blaZ* + *mup* and *ermA* + *ermC* + *blaZ*, respectively. Moreover, the greatest frequency of virulence gene patterns in these two clusters was respectively related to *hla* + *sea* and *hla* + *hlb*. However, the *sec*, *eta*, *tst*, and *pvl* genes were detected in none of the MRSA isolates.

## Discussion

This study aimed to determine the prevalence, virulence genes, and antimicrobial susceptibility of the *dru* types of MRSA with SCC*mec* III isolated from burn wounds in a burn care center in Tehran, Iran. Study results illustrated that the prevalence of MRSA among patients with burn wounds was 69%. This prevalence rate is higher than the rates reported in earlier studies in Iran [20, 29, 30], except for a study in burn centers in Ahvaz which reported a prevalence rate of 80% [31]. Moreover, the prevalence of MRSA in the present study was higher than the rates reported in burn centers in the United States (32%), European countries (26%), and Australia (23%) [32–34]. This difference can be attributed to the differences in infection control policies used in different areas.

Our study also showed that a high proportion of MRSA isolates harbored SCC*mec* type IIIA. Similarly, studies in Iran and other Asian countries reported the high prevalence of SCC*mec* type IIIA [13, 35–37]. SCC*mec* types III and IIIA were also detected in Hungarian and Brazilian clones [36]. MRSA isolates with SCC*mec* types III can act as large reservoirs of enterotoxins and antimicrobial resistance and prevail in communities. Our previous survey shown that the *sea, hla, fib* and *icaA* were most frequent genes encoding virulence factors among MRSA with SCC*mec* type IIIA [4]. Therefore, accurate diagnosis and effective control strategies are essential to minimize their prevalence. Otherwise, they may become prevalent and cause serious consequences in a near future.

None of the MRSA isolates in the present study were resistant to vancomycin. Although MRSA non-resistance to vancomycin in our study supports the effectiveness of this antibiotic in managing MRSA, this antibiotic should be prescribed with great caution in order to prevent the emergence of vancomycin-resistant *S. aureus*.

The prevalence of MRSA resistance to mupirocin in the present study was 68%. However, earlier studies reported lower rates of MRSA resistance to mupirocin. For instance, studies on burn patients in England, India, and Iran reported that this rate was 5.1, 22.7, and 34%, respectively [31, 38, 39]. The use of mupirocin for treating burn wound infections caused by *S. aureus* might have caused resistance to mupirocin among MRSA isolates. Previous studies in the setting of the present study showed *Pseudomonas aeruginosa* as one the main causes of burn wound infection in recent years [40, 41]. Of course, there are no detailed data about the use of mupirocin in the study setting. However, a study reported a substantially high prevalence of mupirocin-resistant MRSA among patients previously treated with mupirocin. Similarly, a high likelihood of mupirocin resistance was observed among patients with *Pseudomonas* infection treated with cefepime [42]. Mupirocin is produced by the Gram-negative bacterium *Pseudomonas fluorescens* and hence, *Pseudomonas* is inherently resistant to mupirocin [43, 44]. Furthermore, the *mupA* gene, which mediates mupirocin resistance in *Pseudomonas*, can move between bacterial isolates and thereby, cause mupirocin resistance in other isolates such as MRSA [45–47].

Our results also indicated dt10di, dt8 h, and dt8i as the most prevalent *dru* types in MRSA with SCC*mec* type IIIA (Fig. 1). The *dru* type dt10di (i.e. cluster I) was mostly prevalent in the first nine months of the study, while the *dru* types dt8 h and dt8i (i.e. cluster II) were mostly prevalent in the last trimester. These findings may be due to the fact that the *dru* types dt8 h and dt8i might have been entered to the study setting in the last trimester or might have been emerged as a result of the polymorphism of dt10ao *dru* type. Unlike our results, a study in Malaysia reported nine *dru* types in SCC*mec* type III, the most prevalent of which was the dt13d *dru* type (41%) [17]. Moreover, a study in Scotland detected 25 *dru* types and 33 *dru* repeats, with the dt10a and dt7c as the most prevalent *dru* types, respectively [16]. The greater number of *dru* types and *dru* repeats in that study compared to our study can be due to the fact that samples in the present study were selected from a single hospital, while samples in that study were selected from different hospitals.

The results of the present study also indicated the higher prevalence of cluster II in the last three months of the study. This may denote the increasing prevalence of this cluster. Moreover, compared with cluster I, cluster II had higher resistance to rifampin. Besides, the presence of the *hlb* gene was more prevalent in cluster II. The most prevalent antimicrobial resistance pattern in cluster I was erythromycin+clindamycin+cotrimoxazole+mupirocin, while the most prevalent antimicrobial resistance pattern in cluster II was erythromycin+clindamycin+cotrimoxazole+rifampin+mupirocin. The latter finding confirms the higher resistance to rifampin in cluster II. Of course, the number of samples in cluster II was small and hence, further studies with larger samples are recommended.

Virulence gene patterns in clusters and *dru* types in the present study showed that the most prevalent virulence gene patterns in cluster I were *hla* + *sea* (39%) and *sea* (12%), while the most prevalent virulence gene pattern in cluster II was *hla* + *hlb* (33.3%) (Data were not presented). Besides, *hla* + *hlb* virulence genes in the dt8i *dru* type were more prevalent than the other *dru* types. Considering the significant roles of these genes in exacerbating skin infections, the prevalence of these strains can complicate the conditions of patients with burn wound infections. Of course, because of the small sample size in cluster II, drawing definitive conclusions in this area is impossible.

## Conclusion

This study shows the high prevalence of SCC*mec* IIIA among MRSA strains isolated from burn wounds in a teaching hospital in Tehran, Iran. These strains are highly resistant to multiple antibiotics. The three most common *dru* types among these strains are dt10i, dt8 h, and dt8i. Clusters with these *dru* types significantly differ from each other respecting their antimicrobial resistance patterns.

## Abbreviations

ATCC: American Type Culture Collection
bp: base pair
CD: Clindamycin
CLSI: Clinical and Laboratory Standards Institute
DAD: Disk Agar Diffusion
*dru*: direct repeat unit
dt: *dru* type
E: Erythromycin
*eta*: exfoliative toxin A
LZD: Linezolid
MIC: Minimum Inhibitory Concentration
MRSA: Methicillin-Resistant *Staphylococcus aureus*
MST: Minimum Spanning Tree
MUP: Mupirocin
PCR: Polymerase Chain Reaction
*pvl*: Panton–Valentine leukocidin
RP: Rifampicin
*S. aureus*: *Staphylococcus aureus*
SCC*mec*: Staphylococcal Cassette Chromosome *mec*
SYN: quinupristin/dalfopristin
TS: trimethoprim/ Sulfamethoxazole
*tst*: toxic shock syndrome toxin
VRSA: Vancomycin-Resistant *Staphylococcus aureus*

## References

[1] Emaneini M, Beigverdi R, van Leeuwen WB, Rahdar H, Karami-Zarandi M, Hosseinkhani F (2018). Prevalence of methicillin-resistant *Staphylococcus aureus* isolated from burn patients in Iran: a systematic review and meta-analysis. J Glob Antimicrob Resist Netherlands.

[2] Rodrigues MVP, Fortaleza CMCB, Riboli DFM, Rocha RS, Rocha C, da Cunha M de LR de S. Molecular epidemiology of methicillin-resistant *Staphylococcus aureus* in a burn unit from Brazil. Burns. Netherlands; 2013 Sep;39(6):1242–1249. 10.1016/j.burns.2013.02.006 23597850

[3] Toscano Olivo TE, de Melo EC, Rocha C, Fortaleza CMCB (2009). Risk factors for acquisition of methicillin-resistant *Staphylococcus aureus* among patients from a burn unit in Brazil. Burns. Netherlands.

[4] Motallebi M, Jabalameli F, Asadollahi K, Taherikalani M, Emaneini M (2016). Spreading of genes encoding enterotoxins, haemolysins, adhesin and biofilm among methicillin resistant Staphylococcus aureus strains with staphylococcal cassette chromosome *mec* type IIIA isolated from burn patients. Microb Pathog. England.

[5] Shahsavan S, Emaneini M, Noorazar Khoshgnab B, Khoramian B, Asadollahi P, Aligholi M (2012). A high prevalence of mupirocin and macrolide resistance determinant among *Staphylococcus aureus* strains isolated from burnt patients. Burns. Netherlands.

[6] Hosseini SS, Niakan M, Saderi H, Motallebi M, Taherikalani M, Asadollahi K (2016). Frequency of genes encoding erythromycin ribosomal methylases among *Staphylococcus aureus* clinical isolates with different D-phenotypes in Tehran. Iran Iran J Microbiol Iran.

[7] Hiramatsu K, Ito T, Tsubakishita S, Sasaki T, Takeuchi F, Morimoto Y, et al. Genomic Basis for Methicillin Resistance in *Staphylococcus aureus.* Infect Chemother. Korea (South); 2013 Jun;45(2):117–36. 10.3947/ic.2013.45.2.117 PMC3780952 24265961

[8] Paterson GK, Harrison EM, Holmes MA (2014). The emergence of mecC methicillin-resistant *Staphylococcus aureus*. Trends Microbiol.

[9] Baig S, Johannesen T, Overballe-Petersen S, Larsen J, Larsen AR, Stegger M (2018). Novel SCC *mec* type XIII (9A) identified in an ST152 methicillin-resistant *Staphylococcus aureus*. Infect Genet Evol.

[10] Asghar AH (2014). Molecular characterization of methicillin-resistant *Staphylococcus aureus* isolated from tertiary care hospitals. Pakistan J Med Sci Pakistan.

[11] Chongtrakool P, Ito T, Ma XX, Kondo Y, Trakulsomboon S, Tiensasitorn C (2006). Staphylococcal cassette chromosome *mec* (SCC*mec*) typing of methicillin-resistant *Staphylococcus aureus* strains isolated in 11 Asian countries: a proposal for a new nomenclature for SCC*mec* elements. Antimicrob Agents Chemother. United States.

[12] Fuchs PC, Kopp J, Hafner H, Kleiner U, Pallua N (2002). MRSA-retrospective analysis of an outbreak in the burn Centre Aachen. Burns. Netherlands.

[13] Namvar AE, Afshar M, Asghari B, Rastegar LA (2014). Characterisation of SCC*mec* elements in methicillin-resistant *Staphylococcus aureus* isolated from burn patients. Burns. Netherlands.

[14] Parhizgari N, Khoramrooz SS, Malek Hosseini SAA, Marashifard M, Yazdanpanah M, Emaneini M (2016). High frequency of multidrug-resistant *Staphylococcus aureus* with SCCmec type III and Spa types t037 and t631 isolated from burn patients in southwest of Iran. APMIS Denmark.

[15] Ryffel C, Bucher R, Kayser FH, Berger-Bachi B (1991). The *Staphylococcus aureus* mec determinant comprises an unusual cluster of direct repeats and codes for a gene product similar to the Escherichia coli sn-glycerophosphoryl diester phosphodiesterase. J Bacteriol United States.

[16] Goering RV, Morrison D, Al-Doori Z, Edwards GFS, Gemmell CG (2008). Usefulness of mec-associated direct repeat unit (dru) typing in the epidemiological analysis of highly clonal methicillin-resistant *Staphylococcus aureus* in Scotland. Clin Microbiol Infect England.

[17] Ghaznavi-Rad E, Goering RV, Nor Shamsudin M, Weng PL, Sekawi Z, Tavakol M (2011). Mec-associated dru typing in the epidemiological analysis of ST239 MRSA in Malaysia. Eur J Clin Microbiol infect dis. Germany.

[18] Bartels MD, Boye K, Oliveira DC, Worning P, Goering R, Westh H (2013). Associations between dru types and SCC*mec* cassettes. PLoS One United States.

[19] Connie R. Mahon, Donald C. Lehman GMJ. Textbook of Diagnostic Microbiology. 5th Edition. Saunders; 2014.

[20] Fatholahzadeh B, Emaneini M, Gilbert G, Udo E, Aligholi M, Modarressi MH (2008). Staphylococcal cassette chromosome mec (SCC*mec*) analysis and antimicrobial susceptibility patterns of methicillin-resistant *Staphylococcus aureus* (MRSA) isolates in Tehran. Iran Microb Drug Resist United States.

[21] Soroush S, Jabalameli F, Taherikalani M, Amirmozafari N, Fooladi AAI, Asadollahi K (2016). Investigation of biofilm formation ability, antimicrobial resistance and the staphylococcal cassette chromosome mec patterns of methicillin resistant *Staphylococcus epidermidis* with different sequence types isolated from children. Microb Pathog. England.

[22] Emaneini M, Jabalameli L, Iman-Eini H, Aligholi M, Ghasemi A, Nakhjavani FA (2011). Multiple-locus variable number of tandem repeats fingerprinting (MLVF) and virulence factor analysis of methicillin resistant *Staphylococcus aureus* SCCmec type III. Polish J Microbiol Poland.

[23] Clinical and Laboratory Standards Institute (CLSI).Available online: https://clsi.org/standards/products/microbiology/ (accessed on 10 September 2017). 2017.

[24] Emaneini M, Bigverdi R, Kalantar D, Soroush S, Jabalameli F, Noorazar Khoshgnab B (2013). Distribution of genes encoding tetracycline resistance and aminoglycoside modifying enzymes in *Staphylococcus aureus* strains isolated from a burn center. Ann Burns Fire Disasters Italy.

[25] Ito T, Kuwahara-Arai K, Katayama Y, Uehara Y, Han X, Kondo Y (2014). Staphylococcal cassette chromosome *mec* (SCC*mec*) analysis of MRSA. Methods Mol Biol United States.

[26] Jarraud S, Mougel C, Thioulouse J, Lina G, Meugnier H, Forey F (2002). Relationships between *Staphylococcus aureus* genetic background, virulence factors, *agr* groups (alleles), and human disease. Infect Immun United States.

[27] Mehrotra M, Wang G, Johnson WM (2000). Multiplex PCR for detection of genes for *Staphylococcus aureus* enterotoxins, exfoliative toxins, toxic shock syndrome toxin 1, and methicillin resistance. J Clin Microbiol. United States.

[28] Nishi J, Miyanohara H, Nakajima T, Kitajima I, Yoshinaga M, Maruyama I (1995). Molecular typing of the methicillin resistance determinant (*mec*) of clinical strains of Staphylococcus based on mec hypervariable region length polymorphisms. J Lab Clin Med United States.

[29] Hoseini Alfatemi SM, Motamedifar M, Hadi N, Sedigh Ebrahim Saraie H. Analysis of Virulence Genes Among Methicillin Resistant *Staphylococcus aureus* (MRSA) Strains. Jundishapur J Microbiol. Iran; 2014 Jun;7(6):e10741. 10.5812/jjm.10741 PMC4217665 25371805

[30] Askari E, Soleymani F, Arianpoor A, Tabatabai SM, Amini A, Naderinasab M (2012). Epidemiology of mecA-methicillin resistant *Staphylococcus aureus* (MRSA) in Iran: a systematic review and meta-analysis. Iran J Basic Med Sci.

[31] Abbasi-Montazeri E, Khosravi AD, Feizabadi MM, Goodarzi H, Khoramrooz SS, Mirzaii M (2013). The prevalence of methicillin resistant *Staphylococcus aureus* (MRSA) isolates with high-level mupirocin resistance from patients and personnel in a burn center. Burns Netherlands.

[32] Guggenheim M, Zbinden R, Handschin AE, Gohritz A, Altintas MA, Giovanoli P (2009). Changes in bacterial isolates from burn wounds and their antibiograms: a 20-year study (1986-2005). Burns. Netherlands.

[33] Hodle AE, Richter KP, Thompson RM (2006). Infection control practices in U.S. burn units. J burn care res. England.

[34] Nimmo GR, Pearson JC, Collignon PJ, Christiansen KJ, Coombs GW, Bell JM (2011). Antimicrobial susceptibility of Staphylococcus aureus isolated from hospital inpatients, 2009: report from the Australian group on antimicrobial resistance. Commun Dis Intell Q Rep Australia.

[35] Aires de Sousa M, Crisostomo MI, Sanches IS, Wu JS, Fuzhong J, Tomasz A, et al. Frequent recovery of a single clonal type of multidrug-resistant *Staphylococcus aureus* from patients in two hospitals in Taiwan and China. J Clin Microbiol. United States; 2003 Jan;41(1):159–63. 10.1128/JCM.41.1.159-163.2003 PMC149637 12517842

[36] Arakere G, Nadig S, Swedberg G, Macaden R, Amarnath SK, Raghunath D (2005). Genotyping of methicillin-resistant *Staphylococcus aureus* strains from two hospitals in Bangalore, South India. J Clin Microbiol United States.

[37] Cirlan M, Saad M, Coman G, Bilal NE, Elbashier AM, Kreft D (2005). International spread of major clones of methicillin resistant *Staphylococcus aureus*: nosocomial endemicity of multi locus sequence type 239 in Saudi Arabia and Romania. Infect Genet Evol Netherlands.

[38] Krishnan PU, Miles K, Shetty N (2002). Detection of methicillin and mupirocin resistance in Staphylococcus aureus isolates using conventional and molecular methods: a descriptive study from a burns unit with high prevalence of MRSA. J Clin Pathol England.

[39] Rudresh MS, Ravi GS, Motagi A, Alex AM, Sandhya P, Navaneeth BV (2015). Prevalence of mupirocin resistance among staphylococci, its clinical significance and relationship to clinical use. J Lab Physicians India.

[40] Heidari H, Emaneini M, Dabiri H, Jabalameli F (2016). Virulence factors, antimicrobial resistance pattern and molecular analysis of Enterococcal strains isolated from burn patients. Microb Pathog England.

[41] Salimi F, Eftekhar F (2014). Prevalence of blaIMP, and blaVIM gene carriage in metallo-beta-lactamase-producing burn isolates of Pseudomonas aeruginosa in Tehran. Turkish J Med Sci Turkey.

[42] Caffrey AR, Quilliam BJ, LaPlante KL (2010). Risk factors associated with mupirocin resistance in meticillin-resistant Staphylococcus aureus. J Hosp Infect England.

[43] Yanagisawa T, Kawakami M (2003). How does Pseudomonas fluorescens avoid suicide from its antibiotic pseudomonic acid?: evidence for two evolutionarily distinct isoleucyl-tRNA synthetases conferring self-defense. J Biol Chem United States.

[44] Sutherland R, Boon RJ, Griffin KE, Masters PJ, Slocombe B, White AR (1985). Antibacterial activity of mupirocin (pseudomonic acid), a new antibiotic for topical use. Antimicrob Agents Chemother. United States.

[45] Patel JB, Gorwitz RJ, Jernigan JA (2009). Mupirocin resistance. Clin Infect Dis United States.

[46] Ramsey MA, Bradley SF, Kauffman CA, Morton TM (1996). Identification of chromosomal location of *mupA* gene, encoding low-level mupirocin resistance in staphylococcal isolates. Antimicrob Agents Chemother. United States.

[47] Driscoll DG, Young CL, Ochsner UA (2007). Transient loss of high-level mupirocin resistance in *Staphylococcus aureus* due to MupA polymorphism. Antimicrob Agents Chemother United States.

